# Health worker densities and immunization coverage in Turkey: a panel data analysis

**DOI:** 10.1186/1478-4491-6-29

**Published:** 2008-12-22

**Authors:** Andrew D Mitchell, Thomas J Bossert, Winnie Yip, Salih Mollahaliloglu

**Affiliations:** 1 Harvard School of Public Health, Boston, Massachusetts, USA; 2 University of Oxford, Oxford, United Kingdom of Great Britain and Northern Ireland; 3 School of Public Health, Ministry of Health, Ankara, Turkey

## Abstract

**Background:**

Increased immunization coverage is an important step towards fulfilling the Millennium Development Goal of reducing childhood mortality. Recent cross-sectional and cross-national research has indicated that physician, nurse and midwife densities may positively influence immunization coverage. However, little is known about relationships between densities of human resources for health (HRH) and vaccination coverage within developing countries and over time. The present study examines HRH densities and coverage of the Expanded Programme on Immunization (EPI) in Turkey during the period 2000 to 2006.

**Methods:**

The study is based on provincial-level data on HRH densities, vaccination coverage and provincial socioeconomic and demographic characteristics published by the Turkish government. Panel data regression methodologies (random and fixed effects models) are used to analyse the data.

**Results:**

Three main findings emerge: (1) combined physician, nurse/midwife and health officer density is significantly associated with vaccination rates – independent of provincial female illiteracy, GDP per capita and land area – although the association was initially positive and turned negative over time; (2) HRH-vaccination rate relationships differ by cadre of health worker, with physician and health officers exhibiting significant relationships that mirror those for aggregate density, while nurse/midwife densities are not consistently significant; (3) HRH densities bear stronger relationships with vaccination coverage among more rural provinces, compared to those with higher population densities.

**Conclusion:**

We find evidence of relationships between HRH densities and vaccination rates even at Turkey's relatively elevated levels of each. At the same time, variations in results between different empirical models suggest that this relationship is complex, affected by other factors that occurred during the study period, and warrants further investigation to verify our findings. We hypothesize that the introduction of certain health-sector policies governing terms of HRH employment affected incentives to provide vaccinations and therefore relationships between HRH densities and vaccination rates. National-level changes experienced during the study period – such as a severe financial crisis – may also have affected and/or been associated with the HRH-vaccination rate link. While our findings therefore suggest that the size of a health workforce may be associated with service provision at a relatively elevated level of development, they also indicate that focusing on per capita levels of HRH may be of limited value in understanding performance in service provision. In both Turkey and elsewhere, further investigation is needed to corroborate our results as well as gain deeper understanding into relationships between health worker densities and service provision.

## Background

Increasing vaccination coverage is an important step towards reducing under-five mortality by two-thirds by 2015, the fourth Millennium Development Goal (MDG). While there have been large reductions in childhood mortality since the second half of the 20^th ^century, over 10 million children still die before the age of five [2]. Vaccine-preventable diseases continue to contribute greatly to this mortality burden, accounting for an estimated 14% of those deaths. Among deaths due to vaccine-preventable diseases, measles alone accounts for around one-third, while pertussis and tetanus combine for another one-third [3]. Since 1974, the World Health Organization's (WHO) Expanded Programme on Immunization (EPI) has been a key tool used by nations to reduce child mortality. Immunizations against measles, diphtheria, pertussis and tetanus (DPT) and polio form the core of all countries' basic EPI package, with other antigens included as a country's level of development and financial resources permit. The importance of a strong EPI framework in reducing child mortality is reflected in one of the indicators of the fourth MDG – the proportion of children vaccinated against measles has been selected as one of the indicators of the fourth MDG. Rate of measles immunization is indicative of the coverage and quality of national health care systems, since most basic health packages in low- and middle-income countries finance vaccinations against measles and DPT [4].

In Turkey, where levels of childhood mortality and morbidity remain above those in many of its neighbouring countries, achieving higher vaccination coverage remains an unmet goal. Turkey is a middle-income country that has experienced substantial economic growth over the past 50 years. As in many other countries with similar development trajectories (e.g. Mexico), it now faces a dual burden of disease wherein communicable diseases continue to weigh down the health of the Turkish people even while the chronic disease burden grows. Infectious diseases account for around 10% of the country's overall disease burden and 80% of childhood deaths [5]. As many children under five die each year (29 per 1000 live births) as middle-aged adults (45–59), and Turkey experiences the eighth highest child mortality rate in the WHO European region [3].

The Turkish Ministry of Health (MOH) has made significant efforts to reduce childhood mortality through increased immunization coverage. Introduced in Turkey in 1980, the government's Expanded Programme of Immunizations includes vaccinations for BCG, polio, DPT, measles, Hepatitis B and tetanus toxoid [6]. Immunizations are provided free of charge by MOH facilities at the primary health care (PHC) level and this delivery system accounts for almost all childhood vaccinations administered in Turkey. Vaccination services are provided primarily by nurses and midwives under the supervision of primary care facility general practitioner physicians. In theory, nurses provide vaccinations only in health facilities, while midwives administer vaccinations both in facilities and in the field. In practice, however, staffing shortages require that their roles be more interchangeable and that PHC officers (akin to male nurses) take part administering vaccinations.

**Figure 1:**
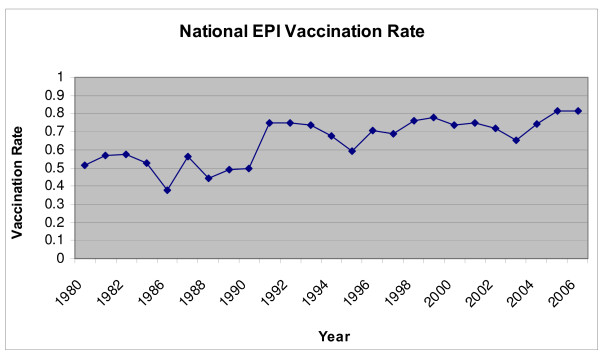
**EPI vaccination rate, 1980–2006**. Source: Immunization Profile – Turkey. .

Vaccination coverage has improved substantially under Turkey's EPI programme. As indicated in Figure 1, the percentage of children receiving EPI vaccinations increased from around 50% in 1980 to around 80% in 2006 (percentages averaged across all antigens). In addition to routine vaccinations provided through the EPI programme, use of National Immunization Days (NIDs) launched since the mid-1990s have helped to significantly increase immunization rates over the past decade. Indeed, the drop in post-neonatal death rates since the 1990s may in part reflect successes surrounding the EPI programme [5].

Nevertheless, improving vaccination coverage remains an important component in reducing the disease burden of Turkey's children. Nationally, Turkey's EPI vaccination rate has hovered between 70% and 80% for almost two decades, and the country's target of 90% complete EPI coverage remains unmet. There also continue to be wide regional differences in vaccination coverage. Lower access to primary care in rural areas is associated with higher rates of childhood mortality from vaccine-preventable diseases, and some previous studies have found vaccination rates in rural areas to be lower than the nationwide average [7-9]. Further, findings from the most recent Demographic and Health Survey (DHS) indicate that in 2003 fewer than 50% of children under five received a full complement of the EPI vaccinations before their first birthday [7]. Indeed, incomplete and uneven coverage may be a contributory factor to outbreaks of measles that seem to occur every three to four years [10] and to persistently elevated levels of childhood mortality more generally.

Recent international research suggests that the size of countries' health workforces can be important in increasing vaccination coverage. The 2004 Joint Learning Initiative's Human Resources for Health report and the 2006 *World health report *focused attention on the many important roles that human resources for health (HRH) play in the functioning of health systems. Findings from the *World health report *were based in part on recent cross-country research examining density of HRH (i.e. number of health workers per population) and health outcomes and service provision, including vaccination coverage. Using 63 country-years of data from 49 countries, Anand and Bärnighausen (2007) examine associations between coverage of three types of vaccines – measles-containing vaccine, DPT and polio – and health worker density. Controlling for GNI per capita, land area and female adult literacy, they find that the combined density of doctors and nurses to population is positively and significantly related to coverage of the three vaccines. When densities are disaggregated by type of health worker, they find that nurse density in particular is positively associated with vaccination coverage, while physician density is not. The authors hypothesize that the opportunity cost for physicians of administering vaccinations is sufficiently high such that an increase in density does not lead to increased vaccination coverage [11].

A second cross-national study finds similar positive relationships. Expanding on a dataset as used by Anand and Bärnighausen (2004), Speybroeck et al. (2006) find a positive relationship between aggregate HRH density and measles coverage [12,13]. Findings from their disaggregated analysis, however, differ from those of Anand and Bärnighausen (2007). Speybroeck et al. find that physician density remains statistically significant with vaccination coverage, while nurse/midwife density does not. The authors hypothesize a number of reasons for differences in findings. Opposite results pertaining to physician density may be due to the generally low levels of physician densities in Anand and Bärnighausen's sample (the implication being that lack of variation in the author's sample inhibited detection of statistical relationships). Non-significance relating to nurses/midwives may be due to greater cross-country heterogeneity in defining these categories of HRH than for physicians (implying greater measurement error undermining true relationships).

While such cross-national studies have begun to construct an evidence base surrounding deployment of health workers and coverage of health services/health outcomes, two major gaps in our knowledge remain. First, little within-country research has been conducted on levels of health workers and health outcomes. As Speybroeck et al. (2006) note, the qualifications, training, classification and roles of health workers vary widely from country to country. Nurses in some countries, for example, may undertake many of the same activities as junior doctors in others. Examining relationships between types of health workers and health service provision at the cross-national level is therefore prone to error. A within-country analysis avoids such limitations and can therefore provide somewhat stronger evidence on these associations.

Second, while previous studies have generated valuable hypotheses on causal relationships between HRH and health outcomes [14], their cross-sectional design inhibits deeper investigation. Just as vaccination coverage may be a function of health worker density, so both vaccination coverage and HRH density may be affected by other unobserved characteristics that enter into the HRH-health relationship. The quality of a country's infrastructure, citizen trust in health institutions and workers, health sector policies and exogenous shocks are all examples of factors that are difficult to measure but may be associated with vaccination coverage and deployment of health personnel. Turkey, for example, experienced a national financial crisis at the end of 2000 and again in early 2001. There are many ways that such a crisis could affect both the demand for and supply of vaccinations. Similarly, a new government came to power in 2002 and instituted a number of reforms related to terms and conditions of HRH employment. These could have affected not only the deployment of personnel but their motivation to undertake preventive activities. Should such unmeasured factors be related to health worker density, the previous studies' empirical estimates may be capturing much more than just the role of health worker levels on vaccination coverage. Additionally, the previous cross-sectional studies provide little insight on how relationships may evolve over time and/or be affected by constantly changing secular forces. Such knowledge could be useful to policy-makers seeking to undertake long-term strategies of raising their country's vaccination coverage.

The present study seeks to answer the questions: Have HRH densities contributed to increasing vaccination rates in Turkey, and what implications do findings hold for raising future vaccination coverage? The analysis takes advantage of a panel dataset to extend prior research on this subject. It offers not only insights into immunization rate variation at any particular time but also changes in immunization rates over time. Panel data analysis also makes it possible to distinguish health worker densities from unobserved (and relatively static) country characteristics that may affect vaccination coverage; this feature addresses the second major limitation of previous research. While it does not purport to make firm declarations on chains of causality between health workers and vaccination coverage, it does provide evidence that goes beyond that provided by cross-sectional studies to date.

## Data and methods

The analysis draws upon three sources of provincial-level data from Turkey that span the period 2000 to 2006. Turkey is composed of 81 administrative provinces within seven broader geographical regions. Provincial-level data on vaccination coverage and levels of public sector human resources are drawn from primary health care statistics published by the Turkish Ministry of Health [15]. Data on provincial population levels, per capita GDP, land area and female adult illiteracy are published by the Turkish Statistical Institute [16].

### Dependent variable

Data on immunizations are collected by the Turkish Ministry of Health based on the national registry system, which records the number of doses administered by the government for a variety of types of vaccinations. Vaccination rates are calculated according to standard administrative methods in which the number of doses of each vaccination is divided by the number of eligible-aged children living in each respective province. The dependent variable is constructed as the mean vaccination rate of the six component immunizations of all vaccinations provided by the national EPI programme (i.e. measles, BCG, Hepatitis B, polio (three doses), DPT (three doses), and tetanus toxoid (two doses) (TT2)). While previous research has focused on relationships between HRH and individual antigens, a composite EPI indicator is justified and more informative in the context of Turkey for two reasons. First, since administration of EPI vaccines is organized and provided by PHC facilities, an average vaccination rate is perhaps more indicative of the effectiveness of that system than relationships with individual antigens. Second, as indicated in Table 1, correlations among the five antigens aimed at communicable diseases are particularly high – ranging from 82% to 99% – while tetanus toxoid exhibits yearly correlations from 60% to 76%. Despite its lower degree of correlation, tetanus typhoid is included in analysis because it (1) is nonetheless part of Turkey's EPI programme and (2) exclusion of this EPI component from analysis does not substantively affect empirical results (results available from authors upon request). A composite EPI indicator therefore adds greater variability and information to the outcome in a way that does not fundamentally alter relationships between individual vaccinations and HRH densities. Indeed, we find empirically that results from EPI analyses do not differ qualitatively from those examining HRH densities and individual vaccination rates (results available from authors upon request).

**Table 1:** Inter-EPI antigen correlations (2000–2005)

	**Measles**	**DPT**	**Polio**	**BCG**	**HBV**
DPT	0.89	1.00			

Polio	0.89	0.99	1.00		

BCG	0.79	0.80	0.80	1.00	

HBV	0.85	0.87	0.87	0.83	1.00

TT2	0.60	0.62	0.62	0.65	0.75

### Independent variables

The choice of independent variables is informed by previous studies and the nature of our dataset. HRH density is measured in two ways: aggregate density of all providers working in public sector primary care facilities (i.e. general practitioners, nurses, midwives and health officers); and disaggregated densities of doctors, nurses/midwives and health officers. Following previous studies, variables on GDP per capita, female adult illiteracy and land area are also included. Data on per capita GDP and female adult illiteracy are limited to the year 2000 – the last year that both variables were calculated as part of Turkey's year 2000 census. Provincial land area is measured in kilometers (squared). Finally, a linear time trend variable (range 0–5) is included, with the inclusion of a squared term to capture temporal non-linearities in EPI vaccination rate evident during the period under study (see Figure 1).

### Estimation strategy

Previous research leads us to hypothesize the following provincial-level model:

Vaccination Rate = *f*(HRH density, time, provincial socioeconomic characteristics, provincial demographic characteristics).

Our theoretical model results in the following estimating equation:

(1) ln⁡(Y1−Y)it=β0+β1ln⁡(HRH/pop)it+β2(TimeTrend)t+β3(TimeTrend)t2+β4ln⁡(GDP/capita)i+β5(FemaleIlliteracy)i+β6ln⁡(LandArea)i+υi+εit

where *Y *is the rate of our composite EPI indicator and *β*_1 _is a (vector of) coefficient(s) relating to *HRH density *in either aggregated or disaggregated form, *i *indexes provinces and *t *indexes years. Equation (1) is a random effects model in which we can explore the relationships between both our time-varying HRH explanatory variables (i.e. health worker densities) and time-invariant provincial characteristics (i.e. GDP per capita, female adult illiteracy and land area). However, such a model also assumes independence between time-varying and time-invariant covariates within each provincial panel (i.e. *Cov*(*X*_*it*_, *α*_*i*_) = 0). Because this assumption may not hold, we also estimate a fixed effects specification of equation (1) (in which *β*_0_, *υ*_*i *_and all time-invariant parameters are absorbed by a new constant *a*_*i*_). We employ a logistic-log functional form to be consistent with – and for the same reasons as – previous research. As described in Anand and Bärnighausen, the logistic functional form of the dependent variables addresses both upper and lower boundedness between 0 and 1 [11].

Our empirical analysis expands upon the base model in equation (1) in two main ways. First, to allow for differing relationships over time between types of health workers, we interact HRH densities with our time trend variable. (We restrict HRH interactions to the time trend main effect and omit interactions with the time trend squared term; our specification is based on our findings that no HRH density-time trend squared term interactions are significant either individually or jointly) This is motivated by our previous observation of the financial crisis and policy changes that took place during our study period. Second, we explore possibilities of different HRH-vaccination relationships among more and less densely populated provinces through stratified analyses that separate provinces above and below the median population density for Turkey. This is motivated by earlier research indicating persistent regional variations in vaccination rates and urban-rural differences in access to PHC.

Given the varying population sizes of our provinces, standard errors are clustered by province to be robust against heteroskedasticity. Such clustering precludes a traditional Hausman specification test to evaluate the random effects model assumption that *Cov*(*X*_*it*_, *α*_*i*_) = 0. Consequently, we conduct an alternative specification test described in [17]. This methodology tests the joint significance of time-varying variables which have been demeaned and entered directly into the random effects estimation; joint significance implies that *Cov*(*X*_*it*_, *α*_*i*_) ≠ 0 and that the random effects estimates are not consistent. All analyses are conducted in STATA 9.0.

## Results

### Descriptive statistics

Overall vaccination rates of EPI immunizations range from 74% to 82% over the study period, for a seven-year average of around 75% (Table 2). Vaccination rates for measles, DPT, polio and BCG are generally higher than the overall EPI average, those of HBV around the average, and those of TT2 the lowest among each type of immunization. There has been an increase in immunization coverage from baseline to endline (e.g. from 0.74 to 0.81 for all EPI immunizations), but the trend is U-shaped, with the lowest point in 2003 rather than a steady increase in vaccination coverage over time (see years 2000 to 2006 of Figure 1).

**Table 2:** Mean vaccination rates, by year

**Year**	**Measles**	**DPT**	**Polio**	**BCG**	**HBV**	**TT2**	**All EPI**
2000	0.84	0.82	0.82	0.79	0.73	0.43	0.74

2001	0.84	0.83	0.83	0.79	0.74	0.43	0.75

2002	0.82	0.78	0.78	0.75	0.74	0.43	0.72

2003	0.74	0.68	0.69	0.72	0.69	0.42	0.66

2004	0.79	0.84	0.83	0.75	0.77	0.47	0.74

2005	0.88	0.89	0.89	0.85	0.84	0.55	0.82

2006	0.90	0.88	0.88	0.84	0.83	0.56	0.81

In terms of human resource indicators, Table 3 indicates that overall nurse and physician densities are at comparable levels – around 2.4 and 2.0 per 10 000 population, respectively – with relatively greater numbers of midwives per 10 000 population (3.7, on average) and fewer PHC health officers. The density of GPs held steady from 2000 to 2002 but then fell by around 2.2 doctors per 10 000 population by 2006. Density of health officers follows a similar pattern but at lower levels. Conversely, nurse and midwife densities have experienced a modest increase over the study period of around one nurse per 3000 population and one midwife per 2000 population.

**Table 3:** Mean HRH densities (per 10,000 population), by year

**Year**	**GPs**	**Nurses/Midwives**	**Other PHC staff**
2000	2.6	5.7	1.4

2001	2.5	6.1	1.3

2002	2.6	5.5	1.3

2003	2.3	5.2	1.1

2004	2.0	6.0	1.2

2005	2.1	6.2	1.3

2006	2.2	6.1	1.2

When overall EPI vaccination rate and HRH densities are stratified into relatively urban and rural provinces (Table 4), two findings emerge. First, the overall vaccination rate during the study period is five percentage points higher in provinces with population densities above the median for the country as a whole. Second, there are slightly different patterns of HRH densities depending upon type of health worker. On the one hand, densities of GPs are roughly the same in high and low population-density provinces. On the other hand, nurse/midwife and health officer densities are higher in relatively rural provinces compared to relatively urban ones. T-tests suggest that differences in densities are statistically significant only for health officers.

**Table 4:** Vaccination Rates and HRH densities – by degree of provincial population density

**Population density**	**Vaccination rate, EPI**	**HRH/10 000 population**		
		
		**GP**	**Nurse/Midwife**	**Health Officer**
High	0.77	2.4	5.7	1.1

Low	0.72	2.3	6.0	1.4

### Regressions

Table 5 presents results from the random and fixed effects models for EPI vaccinations (for comparison purposes, the first column of each random and fixed effects model omits all HRH terms). One province (Duzce) was excluded from regression analysis due to its singularity: it came into existence in 2000, after a major earthquake in 1999. While inclusion of this province did not quantitatively affect regression point estimates/statistical significance, our alternative Hausman tests suggested that significant correlations between our time-varying and -invariant variables were inordinately influenced by this province, suggesting that HRH density-vaccination coverage processes here were fundamentally different than for the rest of Turkey (given the substantial need for HRH and health infrastructure – including vaccines – in this province due to the earthquake emergency, this finding is perhaps not surprising).

**Table 5:** Random and fixed effects estimates of EPI vaccination rates on HRH densities (β coefficients presented; standard errors in parentheses) (N = 560; # provinces = 80)

	**Random effects**					**Fixed effects**			
	
	**Baseline**	**Model I**		**Model II**		**Model I**		**Model II**	
Log HRH density	0.00	0.24*	0.50**	0.00	0.00	0.07	0.29	0.00	0.00
	
	0.00	(0.10)	(0.20)	0.00	0.00	(0.20)	(0.20)	0.00	0.00

Log HRH density * Time Trend	0.00	0.00	-0.11**	0.00	0.00	0.00	-0.12**	0.00	0.00
	
	0.00	0.00	(0.04)	0.00	0.00	0.00	(0.04)	0.00	0.00

Log GP density	0.00	0.00	0.00	0.12	0.35	0.00	0.00	-0.06	0.15
	
	0.00	0.00	0.00	(0.10)	(0.20)	0.00	0.00	(0.10)	(0.20)

Log GP density * Time Trend	0.00	0.00	0.00	0.00	-0.13**	0.00	0.00	0.00	-0.15**
	
	0.00	0.00	0.00	0.00	(0.05)	0.00	0.00	0.00	(0.05)

Log nurse/midwife density	0.00	0.00	0.00	0.06	-0.13	0.00	0.00	0.02	-0.19
	
	0.00	0.00	0.00	(0.09)	(0.20)	0.00	0.00	(0.10)	(0.20)

Log nurse/midwife density * Time Trend	0.00	0.00	0.00	0.00	0.09	0.00	0.00	0.00	0.10
	
	0.00	0.00	0.00	0.00	(0.05)	0.00	0.00	0.00	(0.05)

Log health officer density	0.00	0.00	0.00	0.08	0.36*	0.00	0.00	0.11	0.44*
	
	0.00	0.00	0.00	(0.08)	(0.10)	0.00	0.00	(0.10)	(0.20)

Log health officer density * Time Trend	0.00	0.00	0.00	0.00	-0.097**	0.00	0.00	0.00	-0.11**
	
	0.00	0.00	0.00	0.00	(0.04)	0.00	0.00	0.00	(0.04)

Time trend	-0.31**	-0.29**	-1.04**	-0.29**	-1.60**	-0.30**	-1.16**	-0.30**	-1.84**
	
	(0.05)	(0.05)	(0.30)	(0.05)	(0.40)	(0.05)	(0.30)	(0.05)	(0.40)

Time trend-squared	0.062**	0.059**	0.060**	0.059**	0.055**	0.061**	0.062**	0.061**	0.056**
	
	(0.01)	(0.01)	(0.01)	(0.01)	(0.01)	(0.01)	(0.01)	(0.01)	(0.01)

Log GDP/capita	0.09	0.09	0.10	0.11	0.13	0.00	0.00	0.00	0.00
	
	(0.10)	(0.10)	(0.10)	(0.10)	(0.10)	0.00	0.00	0.00	0.00

Log % adult female illiteracy	-1.44**	-1.28**	-1.30**	-1.26**	-1.30**	0.00	0.00	0.00	0.00
	
	(0.20)	(0.20)	(0.20)	(0.20)	(0.20)	0.00	0.00	0.00	0.00

Log Land area	-0.01	0.02	0.02	0.02	0.03	0.00	0.00	0.00	0.00
	
	(0.07)	(0.06)	(0.07)	(0.06)	(0.07)	0.00	0.00	0.00	0.00

Constant	-0.88	0.58	2.36	0.87	3.70*	1.82	3.36*	1.92	5.16**
	
	(1.20)	(1.30)	(1.60)	(1.50)	(1.90)	(1.20)	(1.50)	(1.40)	(1.80)

R-squared (within)	0.26	0.26	0.30	0.26	0.34	0.26	0.30	0.27	0.35

R-squared (between)	0.67	0.72	0.71	0.73	0.69				

R-squared (overall)	0.50	0.52	0.53	0.53	0.54				

F-test: HRH = 0^†^	0.00	0.00	10.90	6.62	20.30	0.00	5.72	0.23	3.90

P-value			<0.01	0.09	<0.01		<0.01	0.88	<0.01

F-test: GP = GP * Time Trend = 0	0.00	0.00	0.00	0.00	8.41	0.00	0.00	0.00	6.83

P-value					0.02				<0.01

F-test: Nurse/Midwife = Nurse/Midwife * Time Trend = 0	0.00	0.00	0.00	0.00	6.63	0.00	0.00	0.00	2.06

P-value					0.04				0.13

F-test: Health officer = Health officer * Time Trend = 0	0.00	0.00	0.00	0.00	7.18	0.00	0.00	0.00	4.36

P-value					0.03				0.02

F-test p-value: Fixed Effects = 0		0.15	0.16	<0.01	<0.01				

** p < 0.01, * p < 0.05

^†^Includes all main effects and interaction terms, where applicable

In terms of the random effects models, Model I suggests that, on average, aggregate PHC HRH density is positively associated with EPI vaccination coverage during the study period (β = 0.24; p = 0.02). This implies that a 10% increase in aggregate HRH density is associated with about a 2.0% increase in probability of a fully completed EPI vaccination schedule. The model with the interaction term suggests that this overall relationship is characterized by a strongly positive main effect association (β = 0.50) and negative interaction term coefficient (β = -0.11). This suggests positive relationships until the year 2004 (e.g. a 10% increase in aggregate HRH density in 2000 is associated with a 3.3% increase in probability of full EPI vaccination coverage) that turn negative thereafter (e.g. by 2006, the same increase in HRH density is associated with a 1.5% reduction in probability of full EPI vaccination coverage).

Model II provides indications that different categories of HRH may be playing different roles in EPI vaccination coverage. While the non-interacted specification does not find significant HRH-vaccination rate relationships – either among each type of health worker individually or jointly – the interacted specification suggests that two different types of relationships may be at play. On the one hand, GP/health officer densities and their respective interaction terms exhibit the same pattern of relationships as aggregate HRH density in Model I and are jointly significant. On the other hand, a negative main effect nurse/midwife term has been counteracted by a positive association (joint F-test of nurse-midwife density and interaction term p-value = 0.04). Both joint F-tests of no significant HRH density terms in the interacted models are highly significant (p < 0.01).

In terms of control variables, adult female illiteracy has a large and negative association with vaccination coverage, wherein a 10% increase is associated with a more than 40% reduction in probability of fully completed EPI vaccination schedule. This is to be expected, given the well-established micro-level link between education and vaccination coverage [12], including previous research from Turkey [9,18,19]. However, neither GDP per capita nor land area is significantly associated with vaccination coverage. As pointed out by Arah (2007), this might reflect collinearities with other independent variables (e.g. positive associations between per capita GDP and both female literacy and HRH densities) [20]. Time trend main effect coefficients are negative with positive squared term coefficients (both highly significant) – a finding consistent with the descriptive results presented in the last seven years of Figure 1. Together, the explanatory variables account for over one-half of variation in our outcome variable. While much of this variation is between provinces, within-province variation is also substantial, particularly given the relatively few time periods. Further, the inclusion of HRH variables increases within-province R-squared from 0.26 to 0.34, suggesting that as much as one-quarter of the explained variation is associated with HRH densities.

Results from the fixed effects estimation models are consistent with the random effects estimates. Though no HRH coefficients in the non-interacted models are significant, the coefficients from interacted versions of both Model I and Model II remain jointly significant (p < 0.01). The main effect aggregate HRH density in Model I remains positive, though the magnitude is attenuated. In terms of disaggregated densities under Model II, both GP and health officer densities remain significantly related to vaccination rates with positive main effect and negative interaction terms. Interestingly, the magnitude of the negative GP/time interaction term suggests that the initial positive associated disappears by 2002 (by the end of the study period, a 10% increase in GP density is associated with an almost 30% decrease in probability of full vaccination coverage). Nurse/midwife density is no longer significant. As with the random effects analyses, joint F-tests of no HRH effects suggest that the interacted versions of each model are appropriate. As with the random effects estimates, comparison of the interacted version of Model II to the baseline version suggests that HRH densities explain a significant portion of variation in vaccination rates.

Interestingly, specification tests do not reject the appropriateness of the random effects model for Model I, but do reject the appropriateness of the random effects estimates for disaggregated analyses. This suggests that while combined doctor, nurse/midwife and health officer densities are not correlated with unobserved provincial characteristics, one or more of each disaggregated densities are so correlated. In fact, further investigation, in which HRH fixed effects were tested separately by type of health worker, suggested that only GP densities are significantly correlated with unobserved provincial characteristics (results not shown).

We also explored how the vaccination-HRH density relationship may vary by level of provincial population density. We restrict presentation of results to the interacted versions of each model and, to be conservative, the fixed effects specifications. Table 6 presents the results stratified by provincial population density. For provinces falling below median population density (i.e. "rural" provinces), two findings emerge. First, results for aggregate HRH are similar to those for the full sample, with an initial positive relationship turning negative after 2003. Second, the positive association/negative associations appear to stem from differing relationships between GPs and health officers. Health officer density exhibits an overall positive relationship with vaccination rate (non-interacted β = 0.46; p = 0.01). Significant associations with GP density, however, appear to stem from the negative interaction over time.

**Table 6:** Fixed effects estimates of EPI vaccination rates on HRH densities – by low/high provincial population density (β coefficients presented; standard errors in parentheses)

	**Low density**				**High density**			
Log HRH density	0.14	0.44	0.00	0.00	-0.01	0.14	0.00	0.00
	
	(0.30)	(0.30)	0.00	0.00	(0.20)	(0.20)	0.00	0.00

Log HRH density * Time Trend	0.00	-0.15*	0.00	0.00	0.00	-0.097*	0.00	0.00
	
	0.00	(0.06)	0.00	0.00	0.00	(0.04)	0.00	0.00

Log GP density	0.00	0.00	-0.25	0.09	0.00	0.00	0.33	0.37
	
	0.00	0.00	(0.20)	(0.30)	0.00	0.00	(0.20)	(0.30)

Log GP density * Time Trend	0.00	0.00	0.00	-0.15*	0.00	0.00	0.00	-0.15
	
	0.00	0.00	0.00	(0.06)	0.00	0.00	0.00	(0.09)

Log nurse/midwife density	0.00	0.00	-0.02	-0.09	0.00	0.00	-0.04	-0.44
	
	0.00	0.00	(0.20)	(0.20)	0.00	0.00	(0.20)	(0.30)

Log nurse/midwife density * Time Trend	0.00	0.00	0.00	0.04	0.00	0.00	0.00	0.18
	
	0.00	0.00	0.00	(0.05)	0.00	0.00	0.00	(0.09)

Log health officer density	0.00	0.00	0.46*	0.59*	0.00	0.00	-0.30	0.23
	
	0.00	0.00	(0.20)	(0.20)	0.00	0.00	(0.20)	(0.30)

Log health officer density * Time Trend	0.00	0.00	0.00	-0.08	0.00	0.00	0.00	-0.15*
	
	0.00	0.00	0.00	(0.05)	0.00	0.00	0.00	(0.06)

Time trend	-0.31**	-1.35**	-0.33**	-1.94**	-0.30**	-0.99**	-0.31**	-1.57*
	
	(0.07)	(0.40)	(0.07)	(0.50)	(0.07)	(0.30)	(0.07)	(0.60)

Time trend-squared	0.063**	0.064**	0.064**	0.059**	0.059**	0.060**	0.061**	0.052**
	
	(0.01)	(0.01)	(0.01)	(0.01)	(0.01)	(0.01)	(0.01)	(0.01)

Constant	2.14	4.23	2.97	6.51*	1.38	2.46	1.17	3.35
	
	(2.00)	(2.30)	(2.30)	(2.80)	(1.20)	(1.60)	(1.60)	(1.90)

R-squared (within)	0.28	0.34	0.31	0.40	0.25	0.28	0.27	0.34

F-test: HRH = 0^†^	0.00	3.34	2.57	3.76	0.00	3.15	0.97	2.00

P-value		0.05	0.07	<0.01		0.05	0.42	0.09

F-test: GP = GP * Time Trend = 0	0.00	0.00	0.00	6.99	0.00	0.00	0.00	1.42

P-value				<0.01				0.25

F-test: Nurse/Midwife = Nurse/Midwife * Time Trend = 0	0.00	0.00	0.00	0.32	0.00	0.00	0.00	2.19

P-value				0.72				0.13

F-test: Health officer = Health officer * Time Trend = 0	0.00	0.00	0.00	3.48	0.00	0.00	0.00	4.57

P-value				0.04				0.02

** p < 0.01, * p < 0.05

† Includes all main effects and interaction terms, where applicable

A somewhat different picture emerges among Turkey's higher-population density (i.e. "urban") provinces. Unlike in more rural provinces, evidence of an overall aggregate HRH relationship with vaccination rates is marginal and characterized mostly by negative relationships among health officers over time. Instead, there are apparently three different types of relationships: a non-significant relationship with GP density, an initially negative association with nurse/midwife density that becomes positive over time, and an initially positive association with other PHC staff that turns negative over time.

### Robustness

We estimated two alternatives to equation (1) to gauge the robustness of our findings. As previously mentioned, the financial crisis of late 2000/early 2001 raises the possibility that our results are driven not primarily by relationships between HRH densities and vaccination coverage but by forces affecting both. Turkey's macroeconomic crisis, which left many citizens worse off in real economic terms, could have affected the supply of government-provided EPI vaccinations through both HRH densities and other non-HRH channels (e.g. governmental immunization budget cuts leading to reduced availability of vaccinations). On the demand side, documented reductions in health utilization [21] might have spilled over into reduced demand for vaccinations by relegating immunizations to a lower priority in people's health-seeking behaviour. Indeed, the decline in immunization rate from 2001 to 2003 could indicate such a scenario. The HRH density-vaccination rate relationships we have found could therefore reflect primarily independent national-level factors associated with HRH densities but not densities per se (i.e. omitted variable bias).

If the driving force behind our results is the financial crisis (or other temporal factor) operating exclusively through non-HRH, we would expect to find no remaining HRH density-vaccination rate relationship once we include time-fixed effects. Results from the fixed-effects version of this model specification are presented in the first four columns of Table 7 (specification tests, not shown, strongly reject the appropriateness of the random effects model for all specifications). Consistent with our earlier findings, there are no significant HRH density terms in the model versions without time interaction terms. When these interactions are included, however, results tell much the same story as before (HRH densities are interacted with the linear time trend term). We also estimated models interacting HRH densities with each year indicator variable. However, F-tests indicated that the average of these year-specific interaction terms for each category of HRH were no different from the interaction coefficient with the linear time trend interaction. Aggregate HRH density still exhibits a positive main effect/negative interaction term and is jointly significant at p < 0.05. Model II again suggests that GP and health officer densities are the driving force behind this relationship, while we find no significant nurse/midwife relationships.

**Table 7:** Fixed effects estimates of EPI vaccination rates on HRH densities – with fixed time effects (β coefficients presented; standard errors in parentheses)

	**Year fixed effects**				**2001–2006**			
	
	**Model I**		**Model II**		**Model I**		**Model II**	
Log HRH density	-0.11	0.11		0.00	0.56**	0.75**		0.00
	
	(0.20)	(0.20)		0.00	(0.10)	(0.10)		0.00

Log HRH density * Time Trend	0.00	-0.13**		0.00	0.00	-0.077**		0.00
	
	0.00	(0.04)		0.00	0.00	(0.03)		0.00

Log GP density	0.00	0.00	0.10	0.22	0.00	0.00	0.01	0.26*
	
	0.00	0.00	(0.10)	(0.20)	0.00	0.00	(0.07)	(0.10)

Log GP density * Time Trend	0.00	0.00	0.00	-0.12*	0.00	0.00	0.00	-0.12**
	
	0.00	0.00	0.00	(0.05)	0.00	0.00	0.00	(0.03)

Log nurse/midwife density	0.00	0.00	-0.23	-0.34	0.00	0.00	0.29**	0.04
	
	0.00	0.00	(0.10)	(0.20)	0.00	0.00	(0.09)	(0.10)

Log nurse/midwife density * Time Trend	0.00	0.00	0.00	0.07	0.00	0.00	0.00	0.082**
	
	0.00	0.00	0.00	(0.05)	0.00	0.00	0.00	(0.03)

Log health officer density	0.00	0.00	0.02	0.33	0.00	0.00	0.26*	0.54**
	
	0.00	0.00	(0.10)	(0.20)	0.00	0.00	(0.10)	(0.10)

Log health officer density * Time Trend	0.00	0.00	0.00	-0.10*	0.00	0.00	0.00	-0.084**
	
	0.00	0.00	0.00	(0.04)	0.00	0.00	0.00	(0.02)

Year	0.00	0.00	0.00	0.00	-0.30**	-0.85**	-0.30**	-1.44**
	
	0.00	0.00	0.00	0.00	(0.03)	(0.20)	(0.03)	(0.20)

Year-squared	0.00	0.00	0.00	0.00	0.060**	0.060**	0.060**	0.059**
	
	0.00	0.00	0.00	0.00	(0.00)	(0.00)	(0.00)	(0.00)

2001	-0.12	-1.03**	-0.10	-1.53**	0.00	0.00	0.00	0.00
	
	(0.09)	(0.30)	(0.09)	(0.40)	0.00	0.00	0.00	0.00

2002	-0.30**	-2.11**	-0.30**	-3.16**	0.00	0.00	0.00	0.00
	
	(0.10)	(0.50)	(0.10)	(0.80)	0.00	0.00	0.00	0.00

2003	-0.63**	-3.36**	-0.62**	-4.94**	0.00	0.00	0.00	0.00
	
	(0.10)	(0.80)	(0.10)	(1.10)	0.00	0.00	0.00	0.00

2004	-0.17	-3.80**	-0.12	-5.96**	0.00	0.00	0.00	0.00
	
	(0.10)	(1.10)	(0.10)	(1.50)	0.00	0.00	0.00	0.00

2005	0.27*	-4.26**	0.32**	-6.98**	0.00	0.00	0.00	0.00
	
	(0.10)	(1.30)	(0.10)	(1.90)	0.00	0.00	0.00	0.00

2006	0.27*	-5.17**	0.31*	-8.43**	0.00	0.00	0.00	0.00
	
	(0.10)	(1.60)	(0.10)	(2.30)	0.00	0.00	0.00	0.00

Constant	0.47	2.08	0.53	3.52*	5.27**	6.59**	5.97**	8.72**
	
	(1.10)	(1.40)	(1.30)	(1.70)	(0.70)	(0.80)	(0.90)	(1.00)

R-squared (within)	0.37	0.41	0.37	0.44	0.53	0.55	0.54	0.59

F-test: HRH = 0^†^	0.00	6.30	1.34	2.90	0.00	20.40	10.40	13.30

P-value		<0.01	0.27	0.01		<0.01	<0.01	<0.01

F-test: GP = GP * Time Trend = 0			0.00	3.42			0.00	6.72

P-value				0.04				<0.01

F-test: Nurse/Midwife = Nurse/Midwife * Time Trend = 0			0.00	1.89			0.00	11.50

P-value				0.16				<0.01

F-test: health officer = health officer * Time Trend = 0	0.00	0.00	0.00	3.14	0.00	0.00	0.00	9.76

P-value	0.00	0.00		0.05	0.00	0.00		<0.01

F-test: HRH^† ^Fixed Effects = 0	0.76	8.22	11.00	38.10	0.76	8.22	0.01	0.26*

P-value	0.38	0.02	0.01	<0.01	0.38	0.02	(0.07)	(0.10)

** p < 0.01, * p < 0.05

† Includes all main effects and interaction terms, where applicable

Though a fixed year effects model may most thoroughly capture the influence of yearly repercussions, it also reduces implies a within-year and -province interpretation of our HRH density variables. Such a model substantially reduces variation in both our outcome and HRH density and therefore power to detect relationships. We thus re-estimated equation (1) as specified but restricted to the time period 2001 to 2006 – the year 2001 corresponding to the first year that the financial crisis would be expected to affect vaccination coverage and/or HRH densities. These estimates are presented in the last four columns of Table 7. When we omit the year 2000 from analysis, we find that HRH densities in both the non-interacted and interacted models exhibit positive associations with vaccination coverage. That is, though aggregate HRH density exhibits a significantly positive main-effects relationship with vaccination coverage and a significantly negative interaction effect, the overall relationship was positive over the six years (β = 0.56). During this period, then, a 10% increase in aggregate HRH density is associated with a 3.6% increase in probability of full EPI vaccination coverage. Disaggregated analyses suggest that the overall positive relationship stems from nurse/midwife and health officer densities (interestingly, though health officer density continues to exhibit an initially positive/subsequently negative relationship, nurse midwife density exhibits the opposite pattern).

## Discussion

Our study suggests that there are relationships between HRH densities and vaccination rates in Turkey, but our results also paint a complicated picture. Our main findings can be summarized as follows. First, combined PHC staff density (GPs, nurses/midwives and health officers) has been positively associated with provincial-level vaccination rates for EPI immunizations over our study period. We estimate that every 10% increase in aggregate densities is associated with a 2% increase in probability of a fully completed EPI vaccination schedule. Further, this relationship is characterized by an initially positive association that diminished and even disappeared over the study period (by the end of the study period, a 10% increase in aggregate density is associated with a 1.5% decrease in probability of a fully completed EPI vaccination schedule). While these point estimates provide a useful starting point for quantifying HRH density-vaccination coverage relationships, we also emphasize that they should be treated with caution for policy purposes. The limited time frame of analysis and sensitivity of results to model specification suggest that further investigation is warranted to verify our results before basing policy on these findings.

Second, our disaggregated analyses indicate that different categories of health workers exhibit differing relationships with vaccination rates. The initially positive/subsequently negative relationships of the aggregate HRH density analyses appear to be driven primarily by densities of GPs and, to a lesser degree, PHC health officers. Nurse/midwife density, on the other hand, exhibits the opposite relationship (initial negative association followed by positive association over time), though the statistical evidence for this relationship is weaker. The weaker connection between nurse/midwife density at the provincial level is somewhat surprising, given that nurses and midwives are primarily responsible for administering vaccinations.

Third, we find evidence of a distributional dimension in which HRH density-vaccination rate relationships are stronger among Turkey's more rural provinces. In relatively rural provinces (i.e. those with population densities below the national median), findings mirror those for the whole sample. Health officer density, in fact, exhibits a significantly positive overall association during the study period. By contrast, there is less evidence that HRH densities have had bearings on vaccination rates among Turkey's more urban provinces. Instead, only PHC health officer densities have had significant relationships with vaccination coverage, and this is characterized by an initially positive association that turned negative relatively soon thereafter.

Finally, HRH density-vaccination rate relationships after 2000 appear to be markedly different from those during our baseline year. When analyses are restricted to the period 2001 to 2006, nurse/midwife and health officer densities have an overall positive relationship with vaccination rate, while GP density continues to have an initially positive/subsequently negative relationship that results in an overall null association.

What factors may be driving these findings? A first possibility is that changing HRH density-vaccination rate relationships relate to policy changes within the MOH that took place during the study period and directly affected service provision. After a newly elected government came to power in 2002, a number of reforms governing the employment of health personnel were instituted. These included the phasing-out of compulsory service in rural areas for physicians, the introduction of contract-based employment for physicians and nurses with salary incentives to serve in rural areas, and the introduction of a performance-based payment system intended to improve health worker productivity and quality of services.

At the PHC level, performance-based pay rewards the achievement of clinical outputs by PHC facility team leaders (i.e. GPs) and both clinical and preventive outputs achieved by the facility (including immunizations). The changing mix of service provision incentives may have affected HRH density-vaccination rate relationships and these changes may have had negative impacts on the vaccination rate. For GPs, for example, the incentives of performance-based pay to heighten personal clinical productivity may have outweighed those designed to ensure a certain level of facility performance. This could have, in turn, focused their attention away from preventive activities such as immunizations. In such a case, a higher density of GPs could be associated with lower vaccination rates during the latter part of our dataset.

Conversely, findings related to health officers might be a result of contract-based employment. While physicians were generally resistant to serving in rural areas under contract-based employment, it has been a relatively successful incentive among other facility personnel. Between 2004 and 2006, for example, over six times as non-physician health personnel were employed under contract than physicians. Coupled with facility-level performance-based pay incentives to designed to maintain preventive activities, such employment could have motivated non-GPs to focus on administering EPI vaccinations. Provinces with higher densities of health officers would therefore also exhibit higher vaccination coverage. However, it is unclear why nurses and midwives would not react similarly to health officers.

A second possibility is that factors other than employment-related incentives – such as the economic crisis in Turkey in late 2000/early 2001 or the general PHC immunization budget – influenced relationships between HRH densities and vaccination rates. Though MOH policies may have played a role in these relationships, it is striking that exclusion of only the baseline year leads to substantially different results. Given that MOH personnel policies took effect only after 2002, the advent of the financial crisis seems a likely candidate that could have significantly affected density-vaccination relationships.

Through our year fixed effects model, we had earlier considered the possibility that such outside forces might entirely erase evidence of HRH density-vaccination rate relationships. While this does not appear to be the case, it is interesting that the nurse/midwife and health officer densities are unconditionally positive after the year 2000. There are many reasons why this might be, including those that operate directly through HRH channels. During times of economic stress, demand for vaccinations might depend to an even greater degree on promotion of preventive activities by HRH than before. Increases in the government's PHC immunization budget could have facilitated stepping up of such efforts: hovering around TRY 20 million from 1999 to 2002, the budget rose to TRY 45 million by 2004 and over TRY 100 million by 2006 [22]. These budgetary trends also correspond to the nationwide rise in vaccination coverage from its low point in 2003. It is possible that the budget increases permitted more effective promotion of preventive immunization activities, and such promotion was especially crucial during and after the crisis. Indeed, this hypothesis is consistent with previous research from Turkey indicating that follow-up visits from midwives are a determinant of vaccination rates [19].

At the same time, however, it is entirely possible that the financial crisis and/or PHC immunization budget may influenced vaccination coverage, while HRH densities were simply associated with these influences (i.e. omitted variable bias). While the financial crisis may have dampened demand for vaccinations in general, for instance, its effects were likely most pronounced among the poorest of Turkey's citizens. At the same time, more wealthy/less rural provinces tend to have higher levels of both vaccination rates and HRH densities. As a result, we might see a positive relationship between nurse-midwife densities and vaccination coverage. This particular possibility would therefore be one of omitted variable bias rather than an exogenous force operating solely through densities of health workers. The increases in PHC immunization budget could have operated the same way, simply by making the supply of vaccinations more accessible.

Our analysis can be of policy interest both internationally and for Turkey. On the one hand, our results suggest that size of the health workforce may matter to service provision even at relatively elevated levels of development. Positive associations between HRH densities and vaccination rates might be expected at low levels of development in which inadequate levels of personnel are significant barriers to access to care. As a middle-income country possessing relatively much higher levels of health personnel, vaccination rates and development compared to low-income countries, it is not clear that the level of health personnel would continue to be a determinant of vaccination coverage in Turkey. It is interesting, then, that we do find evidence of relationships between HRH density and vaccination rates. While positive relationships are more apparent among Turkey's more rural provinces, income levels in those provinces are still close to the average of all low- and middle-income countries (USD 3700) [23]. This finding therefore suggests that HRH densities might matter for health services even at relatively elevated levels of development, and that Turkey's lessons are relevant for many other developing countries. Though it would be premature to draw strong policy conclusions based on our results alone, we hope that our results encourage further investigation in Turkey to verify these findings. Given the paucity of research relating the health workforce to health and service provision outcomes, endeavors similar to ours would be of great use in other countries, as well.

On the other hand, our findings also suggest that focusing on per capita levels of health personnel may be of limited value in workforce planning designed to achieve health systems objectives. There are a variety of ways that governments typically assess workforce requirements, including needs-based approaches, utilization or demand-based approaches, target-setting and density benchmarking [24].

While the first three approaches make attempts to relate country-specific conditions to size of the health workforce, such as disease profile, demand for services and health worker productivity, they also require more sophisticated modeling and/or deeper data requirements than benchmarking of HRH densities. For these reasons, then, policy-makers often focus on per capita levels of health personnel to gauge the adequacy of a country's health workforce. Indeed, Turkey's MOH has maintained for some time that the size of its physician workforce is inadequate because, compared to its European neighbours, its densities are relatively small [6].

Our findings, however, underscore that the size of a country's workforce is only one part of effective delivery of services. As pointed out by Arah (2007), there are wide variations in HRH densities and vaccination coverage even at the cross-national level [20]. These variations suggest that a variety of other factors – political, health-systems, economic, educational, etc. – may mediate relationships between health personnel density and vaccination coverage and merit future research. Our results are consistent with such a conclusion.

While some of the hypotheses we have offered by way of interpretation are not actionable from a MOH policy perspective (e.g. influence of the financial crisis), others are policy levers directly under the government's control (e.g. incentives of employment policies). A deeper understanding of factors affecting linkages between HRH densities and provision of vaccinations could thus be of great value for future workforce planning in Turkey and countries more generally. Future research on HRH densities and provision of services could therefore benefit greatly from a better understanding of health worker performance. For Turkey's MOH, this might take the form of analyses on the effects of performance-based pay or compulsory service on outcomes.

Finally, our results may be of particular interest to the Turkish MOH in future provision of primary care services in Turkey. The MOH is currently emphasizing the role that primary health care must play in addressing Turkey's disease priorities [25]. The family medicine model emphasizes an approach to care in which GPs lead teams of PHC health workers to provide services. Our findings raise the possibility that different health worker cadres may be able to act as substitutes in provision of immunization services. That health officer density was positively associated with vaccination coverage in higher-density provinces during the entire study period – and nurse-midwife density from 2001 onwards – while positive associations for GP density disappeared over time, is consistent with such substitutability.

While our findings alone are not sufficient to form the basis of related policy decisions, their nuanced nature suggests that a better understanding of potential roles for each team-based approach may be important in helping Turkey improve vaccination coverage and bring its level of childhood mortality more in line with its European neighbors. More generally, it would be useful for the government to understand how its family medicine approach may affect other aspects of service provision through similar avenues of research.

There are two main limitations to our study. First, previous studies have raised concerns about the accuracy of immunization rates reported by routine registry systems. In a study of 45 countries, Murray et al. found that officially reported DPT coverage levels were systematically higher than those from demographic health surveys (DHS) [26]. However, comparison of Turkey's officially reported estimates to those of DHS and international agencies do not suggest systematic reporting bias during the time period under study. WHO/UNICEF estimates of national coverage for measles-containing vaccine, DPT, polio and Hepatitis B from 2000 to 2006, for instance, are virtually identical to nationally-reported figures [27]. Similarly, DHS data from 2003 do not suggest the presence of an upward bias over all EPI vaccinations. Though the official estimate of 68% for DPT is higher than that of the DHS (64%), estimates for polio vaccination are identical and country estimates for BCG, measles and tetanus vaccination are lower than the DHS [1]. This is more suggestive of random measurement error than systematic biases. If so, we would expect this error to attenuate our HRH coefficient estimates towards the null rather than inflate them.

Second, our findings are limited to provincial-level vaccination rates and cannot be directly linked to individual-level outcomes. For instance, our EPI analyses suggest that HRH densities have positive relationships with the odds of administering a full set of immunizations for the population at hand. This is different from the odds of an individual child in that province receiving those vaccinations. Indeed, as highlighted previously, recent DHS data suggests those rates are much lower (less than 50%). Nevertheless, we would expect our outcome rates – number of doses administered per eligible age population – to be correlated with individual-level degree of vaccination schedule completion. Further, our outcomes remain indicative of a health system's capacities to reach its citizens. The policy lessons described earlier therefore remain.

ConclusionAn emerging literature has begun to establish links between human resources for health (HRH) and population health. At the cross-national level, there appear to be positive relationships between HRH densities and vaccination coverage, as well as other indicators of health status. To our knowledge, ours is the first study in the field of health to extend such research within a developing country context and by analysing changes over time. Though our study also suggests that there are relationships between HRH densities and vaccination rates in Turkey during our study period, it paints a more complicated picture than depicted by previous evidence at the international level. Our findings suggest that a deeper understanding of relationships between HRH and health would be of use to policy-makers in Turkey, and should motivate additional within-country research into HRH densities and health worldwide.

## Competing interests

The authors declare that they have no competing interests.

## Authors' contributions

AM performed the statistical analyses and drafted the manuscript. TB participated in developing the research question and drafting the manuscript. WY participated in statistical analyses. SM participated in drafting the manuscript.

## References

[B1] Immunization Profile – Turkey. http://www.who.int/immunization_monitoring/en/.

[B2] United Nations (2006). The Millennium Development Goals Report: 2006.

[B3] Core Health Indicators. Turkey. http://www.who.int/whosis/database/country/compare.cfm?country=TUR&indicator=MortChildBoth&language=english.

[B4] Millennium Development Goals. http://ddp-ext.worldbank.org/ext/GMIS/gdmis.do?siteId=2&contentId=Content_t15&menuId=LNAV01HOME1.

[B5] Ministry of Health of Turkey (2004). National Burden of Disease and Cost Effectiveness Project: Burden of Disease Final Report.

[B6] Ministry of Health of Turkey (2004). Turkey Health Report. Ankara.

[B7] Hacettepe University Institute of Population Studies (2004). Turkey Demographic and Health Survey, 2003.

[B8] Altinkaynak S, Ertekin V, Guraksin A, Kilic A (2004). Effect of several sociodemographic factors on measles immunization in children of Eastern Turkey. Public Health.

[B9] Torun SD, Bakirci N (2006). Vaccination coverage and reasons for non-vaccination in a district of Istanbul. BMC Public Health.

[B10] Guris D, Bayazit Y, Ozdemirer U, Buyurgan V, Yalniz C, Toprak I, Aycan S (2003). Measles epidemiology and elimination strategies in Turkey. J Infect Dis.

[B11] Anand S, Bãrnighausen T (2007). Health workers and vaccination coverage in developing countries: an econometric analysis. Lancet.

[B12] Anand S, Bãrnighausen T (2004). Human resources and health outcomes: cross-country econometric study. Lancet.

[B13] Speybroeck N, Kinfu Y, Dal Poz M, Evans D (2006). Reassessing the relationship between human resources for health, intervention coverage and health outcomes. Background paper prepared for the World Health Report 2006.

[B14] DuBois C-A, McKee M (2006). Cross-national comparisons of human resources for health – what can we learn?. Health Economics, Policy and Law.

[B15] Ministry of Health. http://www.saglik.gov.tr/.

[B16] State Institute of Statistics. http://www.tuik.gov.tr/Start.do.

[B17] Wooldridge JM (2002). Econometric analysis of cross section and panel data.

[B18] Topuzoglu A, Ozaydin GA, Cali S, Cebeci D, Kalaca S, Harmanci H (2005). Assessment of sociodemographic factors and socio-economic status affecting the coverage of compulsory and private immunization services in Istanbul, Turkey. Public Health.

[B19] Ozcirpici B, Sahinoz S, Ozgur S, Bozkurt AI, Sahinoz T, Ceylan A, Ilcin E, Saka G, Acemoglu H, Palanci Y (2006). Vaccination coverage in the South-East Anatolian Project (SEAP) region and factors influencing low coverage. Public Health.

[B20] Arah OA (2007). Health workers and vaccination coverage in developing countries. Lancet.

[B21] World Bank (2003). Turkey: Reforming the Health Sector for Improved Access and Efficency (Volumes I and II). Report No 24358-TU.

[B22] Ministry of Health of Turkey (General and Strategic Planning Directorates) Personal Communication. Ankara.

[B23] World Development Indicators. http://web.worldbank.org/WBSITE/EXTERNAL/DATASTATISTICS/0,,contentMDK:20398986~menuPK:64133163~pagePK:64133150~piPK:64133175~theSitePK:239419,00.html.

[B24] Dreesch N, Dolea C, Dal Poz MR, Goubarev A, Adams O, Aregawi M, Bergstrom K, Fogstad H, Sheratt D, Linkins J (2005). An approach to estimating human resource requirements to achieve the Millennium Development Goals. Health Policy Plan.

[B25] Ministry of Health of Turkey (2006). Family Medicine: The Turkish Model.

[B26] Murray CJ, Shengelia B, Gupta N, Moussavi S, Tandon A, Thieren M (2003). Validity of reported vaccination coverage in 45 countries. Lancet.

[B27] WHO/UNICEF (2007). Review of National Immunization Coverage – Turkey (1980–2006).

